# Risk for In-Hospital Complications Associated with COVID-19 and Influenza — Veterans Health Administration, United States, October 1, 2018–May 31, 2020

**DOI:** 10.15585/mmwr.mm6942e3

**Published:** 2020-10-23

**Authors:** Jordan Cates, Cynthia Lucero-Obusan, Rebecca M. Dahl, Patricia Schirmer, Shikha Garg, Gina Oda, Aron J. Hall, Gayle Langley, Fiona P. Havers, Mark Holodniy, Cristina V. Cardemil

**Affiliations:** ^1^CDC COVID-19 Emergency Response Team; ^2^Epidemic Intelligence Service, CDC; ^3^Office of Population Health, Public Health Surveillance and Research Group, U.S. Department of Veterans Affairs, Washington, D.C.; ^4^U.S. Public Health Service, Rockville, Maryland;^ 5^Division of Infectious Diseases & Geographic Medicine, Stanford University, Stanford, California.

*On October 20, 2020, this report was posted as an *MMWR *Early Release on the *MMWR *website (https://www.cdc.gov/mmwr).*

Coronavirus disease 2019 (COVID-19) is primarily a respiratory illness, although increasing evidence indicates that infection with SARS-CoV-2, the virus that causes COVID-19, can affect multiple organ systems (*1*). Data that examine all in-hospital complications of COVID-19 and that compare these complications with those associated with other viral respiratory pathogens, such as influenza, are lacking. To assess complications of COVID-19 and influenza, electronic health records (EHRs) from 3,948 hospitalized patients with COVID-19 (March 1–May 31, 2020) and 5,453 hospitalized patients with influenza (October 1, 2018–February 1, 2020) from the national Veterans Health Administration (VHA), the largest integrated health care system in the United States,* were analyzed. Using *International Classification of Diseases, Tenth Revision,* *Clinical Modification* (ICD-10-CM) codes, complications in patients with laboratory-confirmed COVID-19 were compared with those in patients with influenza. Risk ratios were calculated and adjusted for age, sex, race/ethnicity, and underlying medical conditions; proportions of complications were stratified among patients with COVID-19 by race/ethnicity. Patients with COVID-19 had almost 19 times the risk for acute respiratory distress syndrome (ARDS) than did patients with influenza, (adjusted risk ratio [aRR] = 18.60; 95% confidence interval [CI] = 12.40–28.00), and more than twice the risk for myocarditis (2.56; 1.17–5.59), deep vein thrombosis (2.81; 2.04–3.87), pulmonary embolism (2.10; 1.53–2.89), intracranial hemorrhage (2.85; 1.35–6.03), acute hepatitis/liver failure (3.13; 1.92–5.10), bacteremia (2.46; 1.91–3.18), and pressure ulcers (2.65; 2.14–3.27). The risks for exacerbations of asthma (0.27; 0.16–0.44) and chronic obstructive pulmonary disease (COPD) (0.37; 0.32–0.42) were lower among patients with COVID-19 than among those with influenza. The percentage of COVID-19 patients who died while hospitalized (21.0%) was more than five times that of influenza patients (3.8%), and the duration of hospitalization was almost three times longer for COVID-19 patients. Among patients with COVID-19, the risk for respiratory, neurologic, and renal complications, and sepsis was higher among non-Hispanic Black or African American (Black) patients, patients of other races, and Hispanic or Latino (Hispanic) patients compared with those in non-Hispanic White (White) patients, even after adjusting for age and underlying medical conditions. These findings highlight the higher risk for most complications associated with COVID-19 compared with influenza and might aid clinicians and researchers in recognizing, monitoring, and managing the spectrum of COVID-19 manifestations. The higher risk for certain complications among racial and ethnic minority patients provides further evidence that certain racial and ethnic minority groups are disproportionally affected by COVID-19 and that this disparity is not solely accounted for by age and underlying medical conditions.

The study population comprised two cohorts of hospitalized adult (aged ≥18 years) VHA patients: 1) those with nasopharyngeal (90%) or other specimens that had tested positive for SARS-CoV-2 by real-time reverse transcription–polymerase chain reaction (RT-PCR) during March 1–May 31, 2020, and 2) those with laboratory-confirmed influenza A or B by rapid antigen assay, real-time RT-PCR, direct or indirect fluorescent staining, or viral culture, during October 1, 2018–February 1, 2020. Patients who received an influenza diagnosis after February 1, 2020, were excluded to minimize the possible inclusion of patients co-infected with SARS-CoV-2. Patients were restricted to those with a COVID-19 or influenza test during hospitalization or in the 30 days preceding hospitalization (including inpatient care at a nursing home). Patients who were still hospitalized as of July 31, 2020, or who were admitted >14 days before receiving testing were excluded from the analysis.

Data from EHRs were extracted from VHA Praedico Surveillance System, a biosurveillance application used for early detection, monitoring, and forecasting of infectious disease outbreaks^†^ and Corporate Data Warehouse. Data included age, sex, race/ethnicity, ICD-10-CM diagnosis codes, hospital admission and discharge date, and, if applicable, date of intensive care unit (ICU) admission and date of death. Thirty-three acute complications (not mutually exclusive) were identified using ICD-10-CM codes from the hospitalization EHR (*2*). Underlying medical conditions were identified using ICD-10-CM codes from inpatient, outpatient, and problem list records from at least 14 days before the specimen collection date (*3*).

Categorical variables were compared using Chi-squared or Fisher’s exact test and continuous variables with Wilcoxon rank sum test. Two-sided p-values <0.05 were considered statistically significant. Among patients with COVID-19, the risk for complications was compared among racial/ethnic groups using log-binomial models, adjusting for age and underlying medical conditions, with White patients as the reference group. Relative risk for complications in patients with COVID-19 compared with those with influenza were estimated using log-binomial models, adjusting for age, sex, race/ethnicity, and underlying medical conditions. To assess bias from seasonality in complications unrelated to influenza or COVID-19, a sensitivity analysis restricted to cases diagnosed during March–May of 2019 (influenza) and March–May of 2020 (COVID-19) was conducted. All analyses were performed using SAS (version 9.4; SAS Institute). The data used in this analysis were obtained for the purpose of public health operations in VHA.^§^ Because no additional analyses were performed outside public health operational activities, the activity was determined to meet the requirements of public health surveillance as defined in 45 CFR 46.102(l)(2), and Institutional Review Board review was not required.

During October 1, 2018–February 1, 2020, 5,746 hospitalized patients received a positive influenza test result and during March 1–May 31, 2020, 4,305 hospitalized patients received a positive SARS-CoV-2 test result. For both groups, testing occurred during the 30 days preceding hospitalization or while hospitalized. A total of 132 patients admitted >14 days before testing were excluded, as were 518 patients who were still hospitalized as of July 31, 2020, leaving 5,453 influenza patients and 3,948 COVID-19 patients for analysis.

Patients with COVID-19 were slightly older than were those with influenza (median = 70 years; interquartile range [IQR] = 61–77 years versus 69 years; IQR = 61–75 years) (p = 0.001), but patients with influenza had higher prevalences of most underlying medical conditions than did those with COVID-19 (Table 1). Black patients accounted for 48.3% of COVID-19 patients and 24.7% of influenza patients; the proportion of Hispanic patients was similar in both groups. The percentage of COVID-19 patients admitted to an ICU (36.5%) was more than twice that of influenza patients (17.6%); the percentage of COVID-19 patients who died while hospitalized (21.0%) was more than five times that of influenza patients (3.8%); and the duration of hospitalization was almost three times longer for COVID-19 patients (median 8.6 days; IQR = 3.9–18.6 days) than that for influenza patients (3.0 days; 1.8–6.5 days) (p<0.001 for all).

**TABLE 1:** Demographics, underlying medical conditions, acute complications, and hospital outcomes among hospitalized patients with COVID-19 (March 1–May 31, 2020) and among historically hospitalized patients with influenza (October 1, 2018–February 1, 2020)* — Veterans Health Administration, United States

Characteristic or condition	No. (%)		P-value
	COVID-19	Influenza	
**Baseline characteristics**			
No. of patients	3,948	5,453	—
Median age at test date, yrs (IQR)	70 (61–77)	69 (61–75)	0.001
Male	3,710 (94.0)	5,116 (93.8)	0.76
**Race/Ethnicity**			
White, non-Hispanic	1,515 (40.4)	3,389 (64.0)	<0.001
Black, non-Hispanic	1,811 (48.3)	1,305 (24.7)	
Other race, non-Hispanic^†^	87 (2.3)	150 (2.8)	
Hispanic or Latino	336 (9.0)	449 (8.5)	
**Underlying medical conditions^§^**			
Asthma	260 (6.9)	565 (10.5)	<0.001
COPD	903 (23.9)	2,261 (42.0)	<0.001
Other lung conditions	534 (14.1)	1,078 (20.0)	<0.001
Blood disorders	123 (3.2)	257 (4.8)	<0.001
Cerebrovascular diseases	468 (12.4)	558 (10.4)	<0.001
Heart disease	1,909 (50.4)	3,068 (57.0)	<0.001
Heart failure	707 (18.7)	1,320 (24.5)	<0.001
Hypertension	2,893 (76.4)	4,082 (75.9)	0.77
Diabetes mellitus	1,873 (49.5)	2,416 (44.9)	<0.001
Renal conditions	1,111 (29.4)	1,468 (27.3)	0.03
Liver diseases	528 (13.9)	687 (12.8)	0.10
Immunosuppression	537 (14.2)	1,033 (19.2)	<0.001
Long-term medication use	451 (11.9)	776 (14.4)	<0.001
Cancer	696 (18.4)	1,341 (24.9)	<0.001
Neurologic/Musculoskeletal	1,602 (42.3)	2,091 (38.9)	<0.001
Endocrine disorders	620 (16.4)	996 (18.5)	0.01
Metabolic conditions	2,525 (66.7)	3,628 (67.5)	0.45
Extreme obesity	333 (8.8)	518 (9.6)	0.18
Any underlying medical condition^¶^	3,541 (93.6)	5,117 (95.1)	0.001
In-hospital complications**			
Respiratory	3,030 (76.8)	5,167 (94.8)	<0.001
Pneumonia	2,766 (70.1)	1,916 (35.1)	<0.001
Respiratory failure	1,834 (46.5)	1,556 (28.5)	<0.001
ARDS	369 (9.3)	29 (0.5)	<0.001
Asthma exacerbation, no./No. (%)^††^	17/260 (6.5)	127/565 (22.5)	<0.001
COPD exacerbation, no./No. (%)^††^	160/903 (17.7)	1,154/2,261 (51.0)	<0.001
Pneumothorax	24 (0.6)	9 (0.2)	<0.001
Cardiovascular	516 (13.1)	911 (16.7)	<0.001
Acute MI/Unstable angina	300 (7.6)	499 (9.2)	0.01
Acute CHF	216 (5.5)	467 (8.6)	<0.001
Cardiogenic shock	36 (0.9)	28 (0.5)	0.02
Hypertensive crisis	53 (1.3)	90 (1.7)	0.23
Acute myocarditis	23 (0.6)	11 (0.2)	0.002
Hematologic	244 (6.2)	135 (2.5)	<0.001
Deep vein thrombosis	131 (3.3)	62 (1.1)	<0.001
Pulmonary embolism	112 (2.8)	72 (1.3)	<0.001
DIC	18 (0.5)	6 (0.1)	0.001
Neurologic	161 (4.1)	116 (2.1)	<0.001
Cerebral ischemia/infarction	125 (3.2)	92 (1.7)	<0.001
Intracranial hemorrhage	27 (0.7)	10 (0.2)	<0.001
Endocrine	79 (2.0)	80 (1.5)	0.05
Diabetic ketoacidosis, no./No. (%)^††^	42/1,873 (2.2)	42/2,416 (1.7)	0.24
Gastrointestinal	77 (2.0)	200 (3.7)	<0.001
Acute hepatitis/liver failure	63 (1.6)	26 (0.5)	<0.001
Renal	1,562 (39.6)	1,434 (26.3)	<0.001
Acute kidney failure	1,541 (39.0)	1,413 (25.9)	<0.001
Dialysis initiation^§§^	120 (3.0)	39 (0.7)	<0.001
Other^¶¶^	1,249 (31.6)	1,258 (23.1)	<0.001
Sepsis	984 (24.9)	1,012 (18.6)	<0.001
Bacteremia	186 (4.7)	100 (1.8)	<0.001
Pressure ulcer	289 (7.3)	144 (2.6)	<0.001
**Hospital outcomes**			
Length of stay, days (IQR)	8.6 (3.9–18.6)	3.0 (1.8–6.5)	<0.001
ICU admission	1,421 (36.5)	961 (17.6)	<0.001
In-hospital mortality	828 (21.0)	190 (3.8)	<0.001

**Abbreviations:** ARDS = acute respiratory distress syndrome; CHF = congestive heart failure; COPD = chronic obstructive pulmonary disease; COVID-19 = coronavirus disease 2019; DIC = disseminated intravascular coagulation; ICU = intensive care unit; IQR = interquartile range; MI = myocardial infarction.

* Data on race or ethnicity were missing for 199 (5.0%) patients with COVID-19 and 160 (2.9%) patients with influenza; data on underlying medical conditions were missing for 163 (4.1%) patients with COVID-19 and 75 (1.4%) patients with influenza; and data on ICU admission was missing for 49 (1.2%) patients with COVID-19. P-values were calculated from Chi-squared or Fisher’s exact test for categorical variables and Wilcoxon rank sum test for continuous variables.

^†^ Among patients with COVID-19, non-Hispanic Other included 22 patients with multiple races documented, 22 American Indians or Alaska Natives, 29 Asians, and 14 Native Hawaiians or other Pacific Islanders. Among patients with influenza, non-Hispanic Other included 47 patients with multiple races documented, 34 American Indians or Alaska Natives, 29 Asians, and 40 Native Hawaiians or other Pacific Islanders.

^§^ Coding of underlying medical conditions was based on *International Classification of Diseases, Tenth Revision,* *Clinical Modification,* (ICD-10-CM) codes and grouping into categories was based primarily on established categorizations from the CDC Hospitalized Adult Influenza Vaccine Effectiveness Network.

^¶^ Excluding hypertension only.

** Complications are not mutually exclusive. Acute complications primarily identified using a list of ICD-10-CM codes published by Chow EJ, Rolfes MA, O'Halloran A, et al. (https://jamanetwork.com/journals/jamanetworkopen/fullarticle/2762991). Complications assessed but not included in Chow et al. include pressure ulcers (ICD-10-CM code L89*) and dialysis initiation (ICD-10-CM codes Z49, Z99.2, Z95.3, and Z91.15 and Current Procedural Terminology codes 90935, 90937, 90940, 90945, 90947, 90999, 0505F, 4045F, 36800, 36810, and 36816).

^††^ Denominator restricted to patients with the underlying medical condition related to the complication (asthma, COPD, or diabetes mellitus).

^§§^ Indication of dialysis during hospitalization without indication of dialysis within the past year.

^¶¶^ Other rare complications reported in <1% of patients with COVID-19 included acute pericarditis (seven, 0.2%), immune thrombocytopenic purpura (seven, 0.2%), Guillain-Barre Syndrome (six, 0.2%), encephalitis (seven, 0.2%), acute disseminated encephalomyelitis and encephalomyelitis (one, <0.1%), thyrotoxicosis (19, 0.5%), hyperglycemic hyperosmolar syndrome (14, 0.4%), acute pancreatitis (16, 0.4%), rhabdomyolysis (83, 2.1%), and autoimmune hemolytic anemia (one, <0.1%).

Among patients with COVID-19, 76.8% had respiratory complications, including pneumonia (70.1%), respiratory failure (46.5%), and ARDS (9.3%). Nonrespiratory complications were frequent, including renal (39.6%), cardiovascular (13.1%), hematologic (6.2%), and neurologic complications (4.1%), as well as sepsis (24.9%) and bacteremia (4.7%); 24.1% of COVID-19 patients had complications involving three or more organ systems. Among COVID-19 patients, nine complications were more prevalent among racial and ethnic minority patients, including respiratory, neurologic, and renal complications, even after adjustment for age and underlying medical conditions (Table 2).

**TABLE 2:** Proportions and adjusted relative risk of selected COVID-19 respiratory and nonrespiratory complications,* by race/ethnicity^†^ — Veterans Health Administration, United States, March 1–May 31, 2020

Complication	White, non-Hispanic (N = 1,515)	Black or African American, non-Hispanic (N = 1,811)		Other race, non-Hispanic^§^ (N = 87)		Hispanic or Latino (N = 336)		P-value**
	No. (%)	No. (%)	aRR (95% CI)^¶^	No. (%)	aRR (95% CI)^¶^	No. (%)	aRR (95% CI)^¶^	
Pneumonia	967 (63.8)	1,322 (73.0)	1.15 (1.10–1.21)	64 (73.6)	1.15 (1.01–1.31)	257 (76.5)	1.21 (1.13–1.31)	<0.001
Respiratory failure	656 (43.3)	860 (47.5)	1.14 (1.06–1.23)	48 (55.2)	1.30 (1.08–1.58)	158 (47.0)	1.13 (0.99–1.28)	0.03
ARDS	118 (7.8)	177 (9.8)	1.25 (1.00–1.57)	15 (17.2)	2.06 (1.24–3.43)	38 (11.3)	1.32 (0.92–1.91)	0.01
Hypertensive crisis	11 (0.7)	33 (1.8)	2.27 (1.13–4.54)	3 (3.4)	4.03 (1.14–14.21)	2 (0.6)	0.87 (0.19–3.90)	0.01
Cerebral ischemia/infarction	29 (1.9)	69 (3.8)	2.42 (1.57–3.74)	2 (2.3)	1.34 (0.33–5.50)	17 (5.1)	3.44 (1.92–6.18)	<0.01
Intracranial hemorrhage	6 (0.4)	15 (0.8)	2.45 (0.88–6.80)	3 (3.4)	10.36 (2.54–42.31)	3 (0.9)	2.69 (0.64–11.25)	0.02
Acute kidney failure	483 (31.9)	845 (46.7)	1.40 (1.28–1.53)	36 (41.4)	1.29 (1.01–1.66)	108 (32.1)	1.06 (0.89–1.26)	<0.001
Dialysis initiation	21 (1.4)	83 (4.7)	2.92 (1.81–4.71)	2 (2.4)	1.47 (0.35–6.16)	9 (2.9)	2.09 (0.97–4.52)	<0.001
Sepsis	306 (20.2)	496 (27.4)	1.42 (1.25–1.61)	29 (33.3)	1.71 (1.25–2.34)	91 (27.1)	1.40 (1.14–1.73)	<0.001

**Abbreviations:** ARDS = acute respiratory distress syndrome; aRR = adjusted risk ratio; CI = confidence interval; COVID-19 = coronavirus disease 2019.

* Complications are not mutually exclusive. Other complications assessed but not statistically different (p-value >0.05) across strata of race/ethnicity included pneumothorax, asthma and chronic obstructive pulmonary disease exacerbation, acute myocardial infarction/unstable angina, acute congestive heart failure, acute myocarditis, cardiogenic shock, deep vein thrombosis, pulmonary embolism, disseminated intravascular coagulation, diabetic ketoacidosis, acute hepatitis/liver failure, bacteremia, and pressure ulcers.

^†^ Data on race/ethnicity were missing for 199 (5.0%) of COVID-19 patients and were excluded from the race/ethnicity stratification.

^§^ Other, non-Hispanic category included 22 patients with multiple races documented, 22 American Indians or Alaska Natives, 29 Asians, and 14 Native Hawaiians or other Pacific Islanders.

^¶^ Separate log-binomial models were run to estimate aRRs for each complication. Pneumonia, respiratory failure, and ARDS models adjusted for age, COPD, asthma, and other lung diseases; hypertensive crisis model adjusted for age, hypertension, heart disease, and heart failure; cerebral ischemia/infarction and intracranial hemorrhage models controlled for age, underlying cerebrovascular diseases, neurologic/musculoskeletal conditions, heart disease, and heart failure; acute kidney failure and dialysis models controlled for age, underlying renal disease, diabetes mellitus, and hypertension.

** P-values calculated from Chi-squared or Fisher’s exact test to compare frequencies of complications among strata of race/ethnicity.

Compared with patients with influenza, patients with COVID-19 had two times the risk for pneumonia; 1.7 times the risk for respiratory failure; 19 times the risk for ARDS; 3.5 times the risk for pneumothorax; and statistically significantly increased risks for cardiogenic shock, myocarditis, deep vein thrombosis, pulmonary embolism, disseminated intravascular coagulation, cerebral ischemia or infarction, intracranial hemorrhage, acute kidney failure, dialysis initiation, acute hepatitis or liver failure, sepsis, bacteremia, and pressure ulcers (Figure). Patients with COVID-19 had a lower risk for five complications (asthma exacerbation, COPD exacerbation, acute myocardial infarction (MI) or unstable angina, acute congestive heart failure (CHF), and hypertensive crisis), although acute MI or unstable angina, acute CHF, and hypertensive crisis were not statistically significant when restricting to patients diagnosed during the same seasonal months.

**FIGURE:**
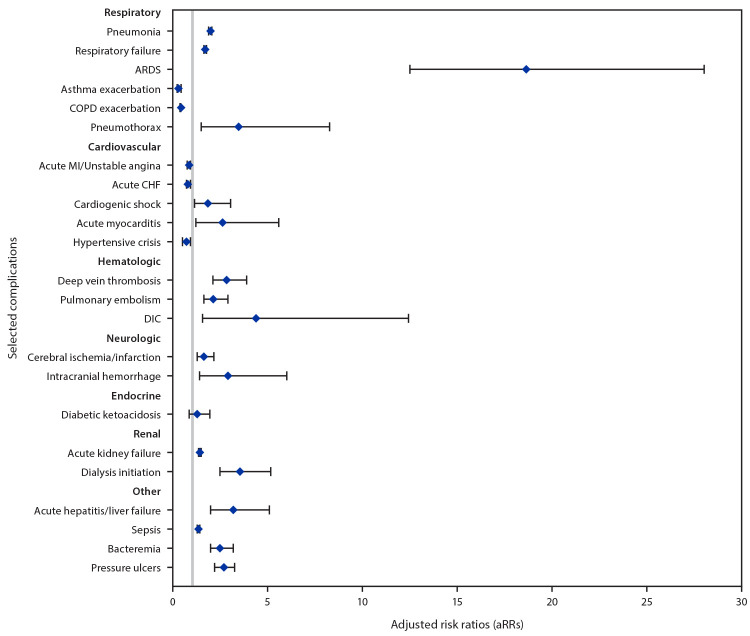
Adjusted relative risk* for selected acute respiratory and nonrespiratory complications in hospitalized patients with COVID-19 (March 1– May 31, 2020), compared with historically hospitalized patients with influenza (October 1, 2018–February 1, 2020) — Veterans Health Administration, United States^†^^,^^§^^,^^¶^ **Abbreviations:** ARDS = acute respiratory distress syndrome; CHF = congestive heart failure; COPD = chronic obstructive pulmonary disease; DIC = disseminated intravascular coagulation; MI = myocardial infarction. * 95% confidence intervals (CIs) indicated with error bars. ^†^ When restricted to patients with influenza during the same seasonal months (March–May), aRRs and 95% CIs for acute MI or unstable angina, acute CHF, and hypertensive crisis were 0.90 (0.74–1.11), 1.03 (0.82–1.28), and 0.75 (0.44–1.29), respectively. ^§^ Dialysis during hospitalization was identified using *International Classification of Diseases, Tenth Revision, Clinical Modification* and current procedural terminology codes, and new initiation of dialysis was determined by excluding patients with indication of dialysis within the past year. ^¶^ Separate crude and adjusted log-binomial models were run for each complication (which were not mutually exclusive). All adjusted models adjusted for age, sex, race/ethnicity, and outcome-specific underlying conditions. Specifically, respiratory complication models controlled for COPD, asthma, and other lung diseases; neurologic complication models controlled for underlying cerebrovascular diseases, neurological/musculoskeletal conditions, heart disease, and heart failure; cardiovascular and hematologic condition models controlled for heart disease, heart failure, renal conditions, diabetes mellitus, and extreme obesity; the acute kidney failure model controlled for underlying renal disease, diabetes mellitus, and hypertension. Complications related to the worsening of a chronic medical condition were restricted to those patients with that underlying medical condition.

## Discussion

Findings from a large, national cohort of patients hospitalized within the VHA illustrate the increased risk for complications involving multiple organ systems among patients with COVID-19 compared with those with influenza, as well as racial/ethnic disparities in COVID-19–associated complications. Compared with patients with influenza, those with COVID-19 had a more than five times higher risk for in-hospital death and approximately double the ICU admission risk and hospital length of stay, and were at higher risk for 17 acute respiratory, cardiovascular, hematologic, neurologic, renal and other complications. Racial and ethnic disparities in the percentage of complications among patients with COVID-19 was found for respiratory, neurologic, and renal complications, as well as for sepsis.

Persons from racial and ethnic minority groups are increasingly recognized as having higher rates of COVID-19, associated hospitalizations, and increased risk for severe in-hospital outcomes (*4*,*5*). Although previous analysis of VHA data found no differences in COVID-19 mortality by race/ethnicity (*4*), in this analysis, Black, Hispanic, and non-Hispanic patients of other races had higher risks for sepsis and respiratory, neurologic, and renal complications than did White patients. The disparities in acute complications among racial and ethnic minority groups could not solely be accounted for by differences in underlying medical conditions or age and might be affected by social, environmental, economic, and structural inequities.^¶^ Elucidation of the reasons for these disparities is urgently needed to advance health equity for all persons.

The risk for respiratory complications was high, consistent with current knowledge of SARS-CoV-2 and influenza pathogenesis (*1*,*6*). Notably, compared with patients with influenza, patients with COVID-19 had two times the risk for pneumonia, 1.7 times the risk for respiratory failure, 19 times the risk for ARDS, and 3.5 times the risk for pneumothorax, underscoring the severity of COVID-19 respiratory illness relative to that of influenza. Conversely, the risk for asthma and COPD exacerbations was approximately three times lower among patients with COVID-19 than among those with influenza.

 The risk for certain acute nonrespiratory complications was also high, including the risk for sepsis and renal and cardiovascular complications. Patients with COVID-19 were at increased risk for acute kidney failure requiring dialysis than were patients with influenza, consistent with previous evidence of influenza- (*2*) and COVID-19–associated (*7*) acute kidney failure. The frequent occurrence and increased risk for sepsis among patients with COVID-19 is consistent with reports of dysregulated immune response in these patients (*8*). The distribution of cardiovascular complications differed between patients with influenza and those with COVID-19; patients with COVID-19 experienced lower risk for acute MI, unstable angina, and acute CHF but higher risk for acute myocarditis and cardiogenic shock. There were no significant differences in occurrence of acute MI, unstable angina, and CHF among patients with COVID-19 or influenza diagnosed during the same months, suggesting potential confounding by seasonal variations in cardiovascular disease.

Other less common (<10%), but often severe complications included hematologic and neurologic complications, bacteremia, and pressure ulcers. Whereas other viruses, like influenza, might cause proinflammatory cytokines and clot formation (*6*), the findings from this study suggest that hematologic complications are a much more frequent complication of COVID-19, consistent with previous reports of COVID-19–related thromboembolic events (*1*,*9*). A New York City study reported that the odds of stroke were 7.6 times higher among COVID-19 patients than among those with influenza (*10*), which is consistent with the present findings of a twofold increase in the risk for cerebral ischemia or infarction. Patients with COVID-19 might be at increased risk for pressure ulcers related to prolonged hospitalizations, prone positioning, or both.

The findings in this report are subject to at least six limitations. First, administrative codes might have limited sensitivity and specificity for capturing conditions and might misclassify chronic conditions as acute. Extreme obesity was defined based solely on ICD-10-CM codes and not body mass index, resulting in potential misclassification and residual confounding. Second, clinician-ordered testing could potentially underestimate some complications in patients with less typical respiratory symptoms. Third, the analysis of racial differences was limited by the small sample size within the non-Hispanic Other race group. Fourth, the generalizability of results might be limited by the diversity and moderate severity among adults of the predominant circulating influenza type/subtype during the period of this analysis (A H3N2 in 2018–2019 and A H1N1 and B in 2019–2020).** Fifth, influenza vaccination or treatments for COVID-19 or influenza that might affect these outcomes were not examined. Finally, this analysis did not adjust for region or facility size or type, and further research is warranted to assess the impact of these factors on the risk for COVID-19 complications. 

Hospitalized adult VHA patients with COVID-19 experienced a higher risk for respiratory and nonrespiratory complications and death than did hospitalized patients with influenza. Disparities by race/ethnicity in experiencing sepsis and respiratory, neurologic, and renal complications, even after adjustment for age and underlying medical conditions, provide further evidence that racial and ethnic minority groups are disproportionally affected by COVID-19. Clinicians should be vigilant for symptoms and signs of a spectrum of complications among hospitalized patients with COVID-19 so that interventions can be instituted to improve outcomes and reduce long-term disability.

**Summary**

**What is already known about this topic?**

Patients hospitalized with COVID-19 are reported to be at risk for respiratory and nonrespiratory complications.

**What is added by this report?**

Hospitalized patients with COVID-19 in the Veterans Health Administration had a more than five times higher risk for in-hospital death and increased risk for 17 respiratory and nonrespiratory complications than did hospitalized patients with influenza. The risks for sepsis and respiratory, neurologic, and renal complications of COVID-19 were higher among non-Hispanic Black or African American and Hispanic patients than among non-Hispanic White patients.

**What are the implications for public health practice?**

Compared with influenza, COVID-19 is associated with increased risk for most respiratory and nonrespiratory complications. Certain racial and ethnic minority groups are disproportionally affected by COVID-19.

## Footnotes

* https://www.va.gov/vetdata/docs/pocketcards/fy20q2.pdf.

†
https://www.oit.va.gov/Services/TRM/ToolPage.aspx?tid=8712 .

§ Access to data for public health activities is covered under the Privacy Act of 1974, System of Records entitled National Patient Databases-VA (121VA10P2) as set forth in 79 FR 8245.

¶ https://www.healthypeople.gov/2020/topics-objectives/topic/social-determinants-of-health.

** https://www.cdc.gov/mmwr/volumes/68/wr/mm6824a3.htm?s_cid; https://www.cdc.gov/mmwr/volumes/69/wr/mm6907a1.htm?s_cid.
